# TAZ as a novel regulator of oxidative damage in decidualization via Nrf2/ARE/Foxo1 pathway

**DOI:** 10.1038/s12276-021-00655-2

**Published:** 2021-09-08

**Authors:** Hai-Fan Yu, Lian-Wen Zheng, Zhan-Qing Yang, Yu-Si Wang, Ting-Ting Wang, Zhan-Peng Yue, Bin Guo

**Affiliations:** 1 College of Veterinary Medicine, Jilin University, Changchun, PR China; 2 Reproductive Medical Center, the Second Hospital of Jilin University, Changchun, PR China

**Keywords:** Reproductive biology, Cell proliferation

## Abstract

TAZ, as a crucial effector of Hippo pathway, is required for spermatogenesis and fertilization, but little is known regarding its physiological function in uterine decidualization. In this study, we showed that TAZ was localized in the decidua, where it promoted stromal cell proliferation followed by accelerated G1/S phase transition via Ccnd3 and Cdk4 and induced the expression or activity of stromal differentiation markers Prl8a2, Prl3c1 and ALP, indicating the importance of TAZ in decidualization. Knockdown of TAZ impeded HB-EGF induction of stromal cell proliferation and differentiation. Under oxidative stress, TAZ protected stromal differentiation against oxidative damage by reducing intracellular ROS and enhancing cellular antioxidant capacity dependent on the Nrf2/ARE/Foxo1 pathway. TAZ strengthened the transcriptional activity of Nrf2 which directly bound to the antioxidant response element (ARE) of Foxo1 promoter region. Additionally, silencing TAZ caused accumulation of intracellular ROS through heightening NOX activity whose blockade by APO reversed the disruption in stromal differentiation. Further analysis revealed that TAZ might restore mitochondrial function, as indicated by the increase in ATP level, mtDNA copy number and mitochondrial membrane potential with the reduction in mitochondrial superoxide. Additionally, TAZ modulated the activities of mitochondrial respiratory chain complexes I and III whose suppression by ROT and AA resulted in the inability of TAZ to defend against oxidative damage to stromal differentiation. Moreover, TAZ prevented stromal cell apoptosis by upregulating Bcl2 expression and inhibiting Casp3 activity and Bax expression. In summary, TAZ might mediate HB-EGF function in uterine decidualization through Ccnd3 and ameliorate oxidative damage to stromal cell differentiation via Nrf2/ARE/Foxo1 pathway.

## Reproduction: Protective protein readies uterus for pregnancy

A protein known to regulate cell proliferation plays a key role in preparing a woman’s uterus for pregnancy, a finding that could inform future treatments for female infertility. A team led by Zhan-Peng Yue and Bin Guo from Jilin University, Changchun, China, examined the role of a co-activator protein called TAZ in decidualization, the process in which the uterine lining changes hormonally and biochemically following ovulation. The researchers showed that TAZ levels build up in the mucosal lining of the uterus, where the protein works with various regulators of the cell cycle to promote the proliferation of connective tissue cells known as stromal cells, which support early embryonic development. The researchers demonstrated that in the face of oxidative stress TAZ helps orchestrate molecular detoxification mechanisms that protect these stromal cells from damage.

## Introduction

In response to implanting embryos, uterine stromal cells proliferate and then differentiate into decidual cells, a process known as decidualization, which is required for pregnancy maintenance^1,2^. Defective decidualization can lead to a range of pregnancy disorders, including embryo miscarriage, recurrent spontaneous abortion, and early pregnancy loss^2,3^. Although numerous genes and signaling pathways have been demonstrated to potentially participate in the process of decidualization, the underlying regulatory mechanisms remain largely unknown.

Transcriptional coactivator with PDZ-binding motif (TAZ), also referred to as WW domain-containing transcription regulator 1 (Wwtr1), is a downstream effector of Hippo pathway that has been established as a principal modulator of organ size, cancer development and stem cell fate, as well as cell proliferation and differentiation^4–6^. Deletion of TAZ in mice reduced litter size and accelerated the lethality of Yes-associated protein (YAP)-deficient embryos^7,8^. In addition, TAZ was detected in spermatogenic and interstitial cells, where its deficiency led to abnormalities in testicular structure and function along with diminished sperm counts and fertility^9^. In zebrafish, TAZ was indispensable for fertilization^10,11^. Although abundant TAZ protein has been found in the nuclei of human endometrial stromal cells^12^, its physiological significance in decidualization has not been reported.

Oxidative stress (OS) is characterized by the disequilibrium between reactive oxygen species (ROS) generation and antioxidant defense and is involved in the pathogenesis of female infertility^13,14^. Accumulating evidence has demonstrated that OS decreases the number of embryos implanted and impairs uterine decidualization, and it is associated with a variety of reproductive disorders, such as endometriosis, recurrent pregnancy loss, and preeclampsia^13–15^. In the context of OS, elevated TAZ protein was transiently observed in HEK 293T cells^16^, implying that TAZ might function as a redox sensor. However, there is limited information regarding whether TAZ ameliorates oxidative damage to decidualization.

The present study suggested the importance of TAZ in uterine decidualization through Ccnd3 and Cdk4 in response to HB-EGF. Furthermore, TAZ might protect stromal differentiation from oxidative damage by restoring cellular antioxidant capacity dependent on the Nrf2/ARE/Foxo1 pathway and preventing mitochondrial dysfunction and cell apoptosis under OS.

## Materials and methods

### Uterine tissue collection

Mouse uteri from days 1–8 of pregnancy and undergoing artificial decidualization were gathered as described previously^17^. All animal experimental procedures were approved by the Committee for the Ethics on Animal Care and Use of Jilin University (SY201905031).

### In situ hybridization

In situ hybridization was performed as depicted previously^17^. Briefly, a TAZ cRNA probe was labeled with digoxigenin and then used for hybridization in frozen sections. After incubation with sheep anti-digoxigenin antibody conjugated to alkaline phosphatase, sections were visualized with BCIP/NBT followed by counterstaining with methyl green. The TAZ primers used for in situ hybridization were listed in Supplementary Table 1.

### In vitro decidualization

In vitro decidualization was performed by replenishing estradiol-17β (10 nM, Sigma) plus progesterone (1 mΜ, Sigma) in uterine stromal cells obtained on the 4th day of pregnancy as depicted previously^17^.

### Immunofluorescence

Decidual cells obtained on day 7 of pregnancy were seeded on glass coverslips to perform immunofluorescence as depicted previously^17^. Briefly, after fixation with 4% cold paraformaldehyde, the cells were incubated overnight with antibody against TAZ (1:100, Thermo Fisher Scientific) followed by the addition of goat anti-rabbit Alex Fluor 488 conjugated antibody (1:1000, Invitrogen) and DAPI counterstaining of the nuclei. Images were obtained by confocal microscopy.

### Western blotting

Proteins from uteri or stromal cells were separated by SDS-PAGE and then transferred onto PVDF membranes. After blocking with 5% skim milk, the membranes were probed overnight with antibodies against TAZ (1:1000, Cell Signaling Technology), phospho-TAZ (Ser89, 1:1000, Cell Signaling Technology), phospho-large tumor suppressor kinase (phospho-LATS, Thr1079, 1:1000, Cell Signaling Technology), nuclear factor erythroid 2-related factor 2 (Nrf2, 1:1000, Proteintech), forkhead box O1 (Foxo1, 1:1000, Proteintech) or Gapdh (1:5000, Proteintech) at 4 °C followed by incubation with HRP-conjugated secondary antibody (1:5000). Signals were visualized with an ECL chemiluminescent kit.

### Real-time PCR

The expression levels of TAZ, prolactin family 8, subfamily a, member 2 (Prl8a2), prolactin family 3, subfamily c, member 1 (Prl3c1), cyclin A1 (Ccna1), Ccnb1, Ccnb2, Ccnd1, Ccnd3, Ccne1, cyclin-dependent kinase 1 (Cdk1), Cdk2, Cdk4, Cdk6, caspase 3 (Casp3), B cell leukemia/lymphoma 2 (Bcl2), Bcl2-associated X protein (Bax), Nrf2 and Foxo1, as well as mitochondrial DNA (mtDNA) copy number, were determined by real-time PCR analysis using a Roche LightCycler 96 Detection System as described previously^15^. The primers used for TAZ, Nrf2 and Foxo1 were listed in Supplementary Table 1, and the other primers were previously described^15,18^.

### Plasmid construction and transfection

Plasmids overexpressing TAZ variant TAZa and TAZb were constructed as described previously^17^, and the primer sequences were provided in Supplementary Table 1. Transfection of this overexpression plasmid was performed with Lipofectamine 3000 according to the manufacturer’s protocol. After introduction of TAZ overexpression plasmid, stromal cells were collected at 12, 24, and 48 h in the absence or presence of estradiol-17β plus progesterone. For further analysis, cells were transfected with TAZ overexpression plasmid and then exposed to 100 μM H_2_O_2_ in the absence or presence of superoxide dismutase (SOD) inhibitor diethyldithiocarbamate (DDC, 10 μΜ, Sigma), catalase (CAT) inhibitor 3-amino-1:2:4-triazole (ATZ, 1 mM, Sigma), glutathione peroxidase (GPX) inhibitor mercaptosuccinic acid (MS, 300 μΜ, Sigma), glutathione reductase (GR) inhibitor 1,3-bis (2-chloroethyl)-1-nitrosourea (BCNU, 20 μΜ, Sigma), GSH synthesis inhibitor buthionine sulfoximine (BSO, 10 μΜ, Sigma), mitochondrial respiratory chain complex I inhibitor rotenone (ROT, 2 μΜ, MCE), mitochondrial respiratory chain complex III inhibitor antimycin A (AA, 2 μΜ, Abcam), Nrf2 inhibitor ML385 (10 μM, MCE) or Foxo1 inhibitor AS1842856 (1 μM, MCE) under in vitro decidualization.

### RNA interference

The following small-interfering RNAs (siRNAs) targeting TAZ were synthesized by GenePharma: CCCUCUUCAACUCUGUCAUTT (siRNA 1), CCACUGGCCAGAGAUACUUTT (siRNA 2), and GCUCAGAUCCUUUCCUCAATT (siRNA 3). Control siRNA (negative control) sequence was described previously^17^. After introduction of TAZ siRNA into stromal cells using the aforementioned Lipofectamine 3000 protocol, the cells were harvested at 12, 24, and 48 h in the absence or presence of estradiol-17β plus progesterone. For further analysis, cells were treated with TAZ siRNA followed by the addition of recombinant HB-EGF protein (rHB-EGF, 100 ng/ml, R&D Systems). In addition, TAZ siRNA-transfected stromal cells were exposed to 100 μM H_2_O_2_ for 4 h with/without the NADPH oxidase (NOX) inhibitor apocynin (APO, 1 μM, MCE) under in vitro decidualization.

### Cell proliferation

Twenty-four hours after introduction of TAZ overexpression plasmid or siRNA into stromal cells in the absence or presence of rHB-EGF, cells were supplemented with MTS reagent (Promega) and incubated for 2–4 h. The absorbance was measured with multimode reader.

### Cell cycle analysis

After introduction of TAZ overexpression plasmid or siRNA into stromal cells for different time courses in the absence or presence of rHB-EGF, cells were fixed, stained with PI/RNase staining buffer (BD Biosciences) and then analyzed by flow cytometry as described previously^15^.

### Alkaline phosphatase (ALP) activity assay

After different treatments, stromal cells were lysed, and ALP activity was measured in accordance with the corresponding assay kit (Beyotime).

### Determination of ROS level

After transfection with TAZ siRNA or overexpression plasmid and then exposure to H_2_O_2_ in the absence or presence of ML385 or AS1842856 during in vitro decidualization, cells were incubated with the fluorescent probe DCFH-DA (Beyotime, 20 μM), dihydroethidium (Beyotime, 10 μM), or MitoSOX Red mitochondrial superoxide indicator (Invitrogen, 5 μM) at 37 °C for different times. The cells were analyzed with multidetection microplate reader or flow cytometry to determine the levels of intracellular ROS, O_2_^−^ and mitochondrial O_2_^−^.

### Measurement of pro- and anti-oxidant parameters

After treatment, proteins were extracted to assess the contents of malondialdehyde (MDA) and reduced glutathione (GSH) as well as GSH/oxidized glutathione (GSSG) ratio and to determine the activities of SOD, CAT, GPX, GR and NOX in accordance with the corresponding assay kit (Beyotime or Solarbio).

### Measurement of mitochondrial membrane potential and ATP content

After treatment, stromal cells were incubated with the JC-1 fluorescent probe and then analyzed by flow cytometry using a mitochondrial membrane potential assay kit (Beyotime). In addition, ATP content was calculated by the corresponding assay kit (Beyotime).

### Assessment of mitochondrial respiratory chain complex I, II and III activities

After transfection with TAZ siRNA or overexpression plasmid and then exposure to H_2_O_2_ under in vitro decidualization, the activities of mitochondrial respiratory chain complexes I, II and III were calculated by the corresponding assay kit (Solarbio).

### Cell apoptosis analysis

After treatment, cell apoptosis was evaluated by flow cytometry with an Annexin V-FITC apoptosis detection kit according to the manufacturer’s instructions (Beyotime). In addition, Casp3 activity was determined with the corresponding assay kit (Beyotime).

### Dual-luciferase reporter gene assay

After Foxo1 promoter sequences (+217 to +416) containing the Nrf2/ARE-binding site were inserted into pGL4.18 vector and then cotransfected with TAZ overexpression plasmid with/without Nrf2 inhibitor ML385, luciferase activity was measured with dual-luciferase reporter gene assay kit (Beyotime). In addition, pARE-luc (Beyotime) or 8xGTIIC-luciferase plasmid (Addgene) was introduced into stromal cells to assess Nrf2 or TAZ-TEAD (TEA domain) transcriptional activity. The pRL-SV40 plasmid (Beyotime) was used for data normalization.

### Statistical analysis

All data were analyzed with the SPSS19.0 software program. Significant difference between two groups was compared with independent-samples *t* tests. Multiple comparisons were evaluated with one-way ANOVA. All values were shown as the means ± SEM. *P* < 0.05 was considered statistically significant.

## Results

### Location and expression of TAZ in pregnant uteri

**Fig. 1:**
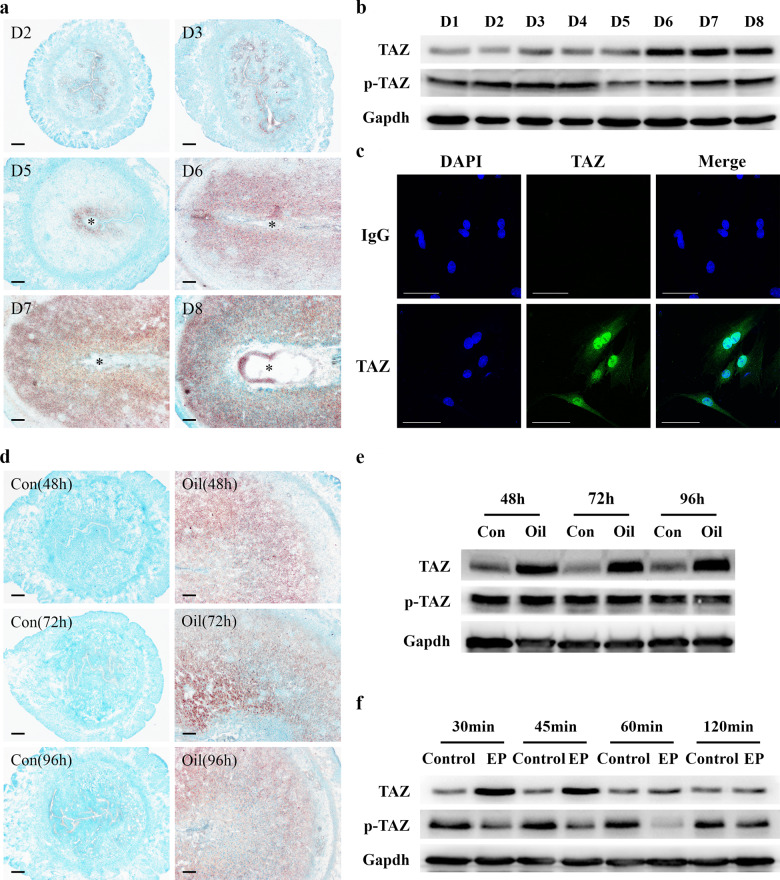
TAZ expression in uteri and decidual cells. **a** Localization of uterine TAZ mRNA on days 2, 3, and 5–8 of pregnancy as determined by in situ hybridization. Scale bar, 60 μm. Asterisks indicate the embryo. **b** Western blotting analysis of TAZ protein in uteri during early pregnancy. TAZ, total TAZ protein; p-TAZ, phospho-TAZ protein. **c** Immunofluorescence analysis showed the marked nuclear localization of TAZ protein in decidual cells. **d** Localization of uterine TAZ mRNA under artificial decidualization. **e** Western blotting analysis of TAZ protein under artificial decidualization. Con, uninjected uterine horn, which served as the control; Oil, oil-induced decidualization. **f** Western blotting analysis of TAZ protein during in vitro decidualization. EP estrogen plus progesterone.

To investigate the relevance between TAZ and early pregnancy events, in situ hybridization was applied to localize TAZ mRNA in pregnant uteri. The results showed that TAZ mRNA was distributed in luminal and glandular epithelia from days 1 to 3 of pregnancy, and exhibited a weak signal in the uteri at day 4 of pregnancy (Fig. 1a). With the onset of implantation, TAZ mRNA was dramatically increased in the subluminal stroma surrounding the implanting blastocyst, but no visible signal was detected at the interimplantation site (Fig. 1a). From days 6 to 8 of pregnancy, uterine decidua showed an apparent signal for TAZ mRNA (Fig. 1a). Real-time PCR and Western blotting analyses verified abundant TAZ mRNA and total protein accumulation in uteri on days 6–8 of pregnancy, but phosphorylated TAZ level was obviously reduced (Fig. 1b, Supplementary Fig. 1a). Immunocytochemistry showed marked nuclear localization of TAZ protein in decidual cells (Fig. 1c). In addition, the mRNA and protein levels of TAZ in decidual cells were higher than those in nondecidualized stromal cells, but TAZ phosphorylation level was decreased (Supplementary Fig. 1b, c).

### TAZ expression in artificially induced decidual issues and cells

To confirm the potential involvement of TAZ in decidualization, we employed in vivo- and in vitro-induced decidualization models. Under oil-infused artificial decidualization, TAZ mRNA signal was mostly localized to decidualizing stromal cells but was not visible in the uninjected control uteri (Fig. 1d). Quantitative analysis of TAZ revealed elevated mRNA and total protein levels in oil-treated uteri, whereas TAZ phosphorylation level remained steady (Fig. 1e, Supplementary Fig. 1d). Moreover, after stromal cells were induced to decidualize, the expression of TAZ mRNA and total protein exhibited a noticeable increase for 30 and 45 min followed by relatively steady expression at 60 and 120 min, whereas TAZ phosphorylation level was decreased, except at 120 min, when it was indistinguishable from that of the control (Fig. 1f, Supplementary Fig. 1e).

### TAZ function in decidualization

**Fig. 2:**
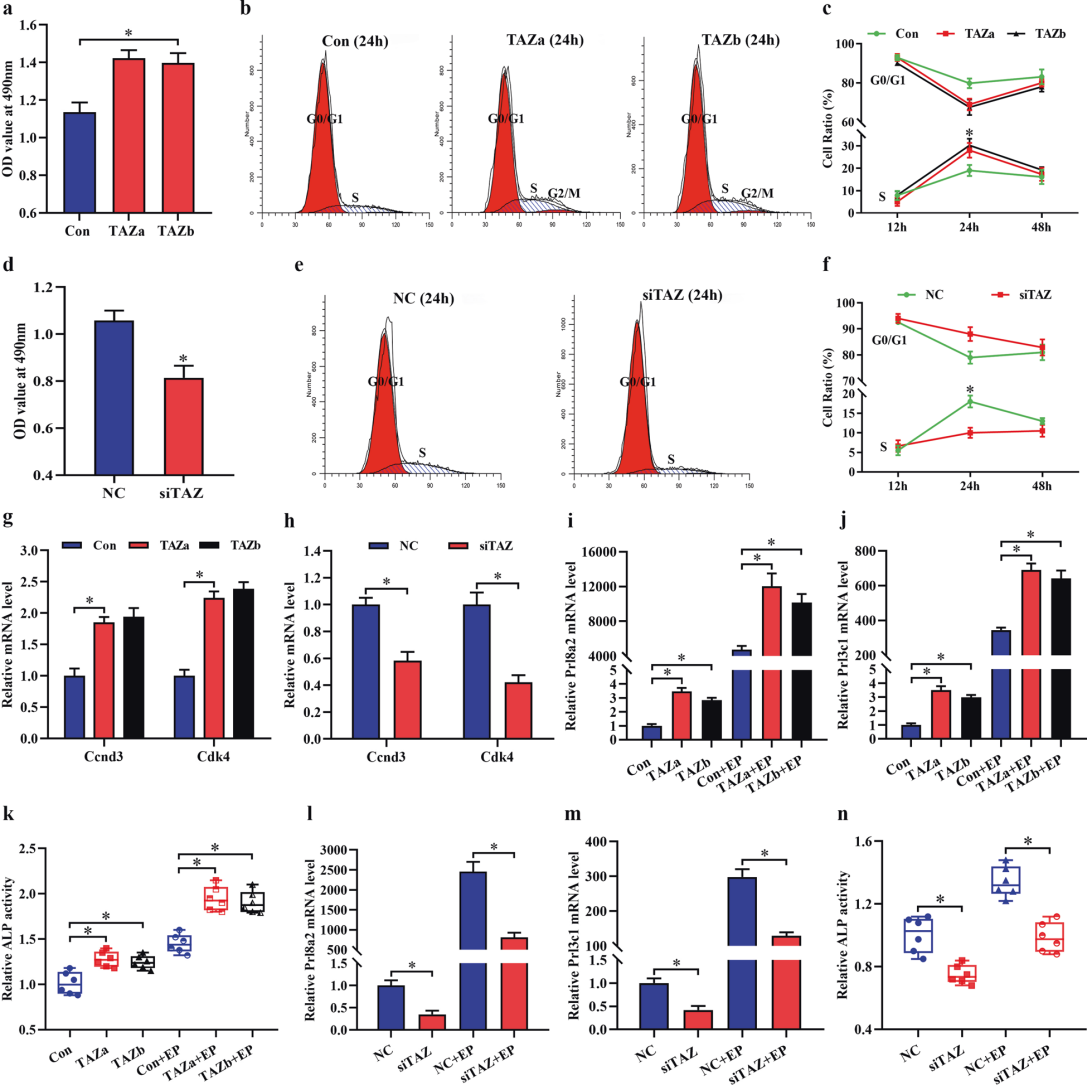
The role of TAZ in uterine decidualization. **a** MTS analysis of stromal cell proliferation at 24 h after introduction of TAZ overexpression plasmid. Con, empty pcDNA3.1 vector; TAZ, TAZ overexpression plasmid. Data are shown the mean ± SEM. Asterisks denote significance (*P* < 0.05). **b**, **c** Flow cytometry analysis of cell cycle distribution after introduction of TAZ overexpression plasmid for 12, 24, and 48 h. **d** MTS analysis of stromal cell proliferation after introduction of TAZ siRNA for 24 h. NC, negative control; siTAZ, TAZ siRNA. **e**, **f** Flow cytometry analysis of cell cycle distribution after introduction of TAZ siRNA for 12, 24 and 48 h. **g**, **h** Regulation of TAZ overexpression or silencing on Ccnd3 and Cdk4 expression at 24 h. **i–k** Constitutive activation of TAZ promoted the expression of Prl8a2 and Prl3c1 and ALP activity at 48 h with/without estrogen and progesterone. **l–n** Knockdown of TAZ markedly repressed Prl8a2 and Prl3c1 expression and ALP activity at 48 h with/without estrogen and progesterone.

Considering the high expression of TAZ in decidual cells, we hypothesize that TAZ is important to uterine decidualization, where stromal cells undergo extensive proliferation and differentiation. To test this hypothesis, TAZ function in stromal cell proliferation was appraised. After introduction of TAZ overexpression plasmid, which dramatically increased the corresponding mRNA and total protein levels and TAZ-TEAD transcriptional activity along with slight elevation for TAZ phosphorylation, stromal cell proliferation rate exhibited an obvious enhancement (Fig. 2a, Supplementary Fig. 2a–c). As indicated by flow cytometry analysis, overexpression of TAZ resulted in an accelerated G1 to S phase transition at 24 h but not at 12 or 48 h (Fig. 2b, c). In contrast, after introduction of TAZ siRNA 2 and 3, which effectively repressed TAZ mRNA and protein levels and weakened TAZ-TEAD transcriptional activity concomitant with notable inhibitory effects for TAZ siRNA 3, stromal cell proliferation was attenuated and G1/S phase transition was stalled at 24 h (Fig. 2d–f, Supplementary Fig. 2d–f). Then, the expression of key cell cycle regulators, namely, cyclins (Ccns) and cyclin-dependent kinases (Cdks), were measured. Constitutive activation of TAZ upregulated the expression of Ccnd3 and Cdk4, while silencing TAZ led to the opposite effect (Fig. 2g, h). TAZ did not seem to have a distinct impact on the mRNA levels of Ccna1, Ccnb1, Ccnb2, Ccnd1, Ccne1, Cdk1, Cdk2, or Cdk6 (data not shown).

To determine the significance of TAZ in stromal cell differentiation, we assessed its regulation of stromal cell differentiation markers Prl8a2 and Prl3c1 expression and ALP activity^17,19^. Sustained activation of TAZ strengthened the levels of Prl8a2 and Prl3c1 mRNA expression as well as ALP activity regardless of the existence or not of estrogen and progesterone (Fig. 2i–k). In contrast, knockdown of TAZ markedly repressed Prl8a2 and Prl3c1 expression and weakened ALP activity (Fig. 2l–n).

### TAZ mediated HB-EGF function in decidualization

**Fig. 3:**
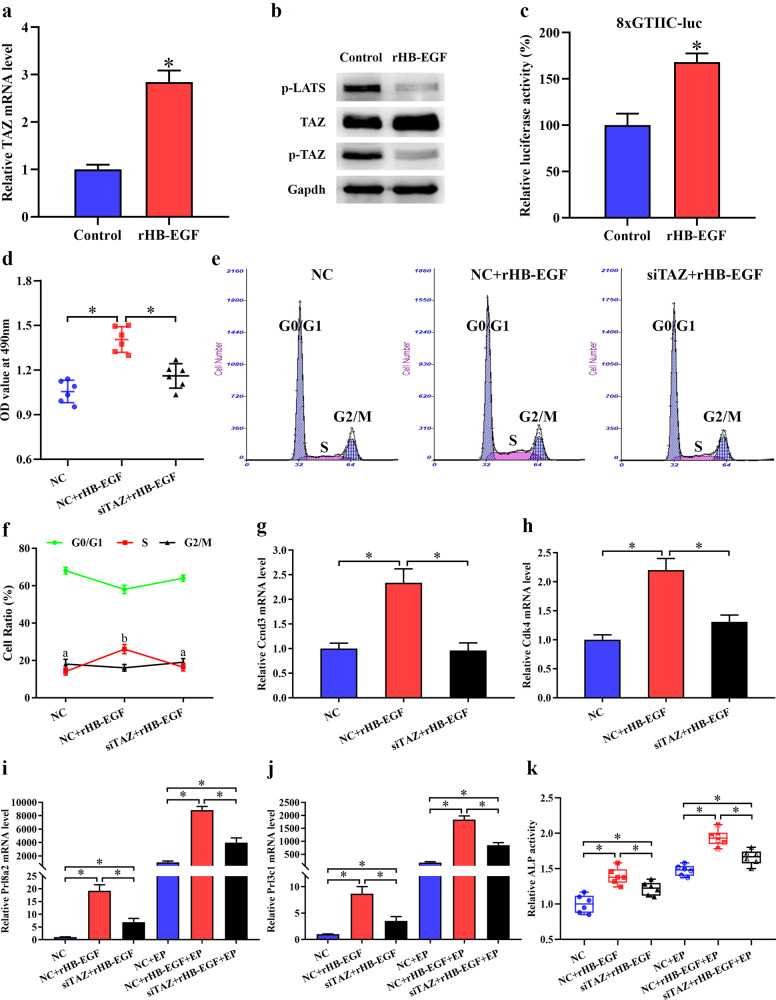
TAZ mediated HB-EGF function in decidualization. **a** TAZ expression after supplementation with rHB-EGF for 48 h. **b** Regulation of HB-EGF on the expression of TAZ and phospho-LATS protein. p-LATS, phospho-LATS protein. **c** Effect of HB-EGF on TAZ-TEAD transcriptional activity. **d** Knockdown of TAZ antagonized HB-EGF-induced stromal cell proliferation. **e**, **f** Flow cytometry analysis showed that TAZ mediated HB-EGF function in the regulation of cell cycle. **g**, **h** Knockdown of TAZ blocked the upregulation of Ccnd3 and Ckd4 that had been induced by HB-EGF. **i–k** TAZ partially attenuated the induction of HB-EGF on Prl8a2 and Prl3c1 expression and ALP activity.

Heparin-binding EGF-like growth factor (HB-EGF) is essential for uterine decidualization^20,21^. Supplementation with exogenous rHB-EGF facilitated the expression of TAZ mRNA and total protein concomitant with an apparent decrease in TAZ phosphorylation (Fig. 3a, b). LATS is required for phosphorylation of TAZ which is retained in the cytoplasm for proteasomal degradation^5,22^. Under in vitro decidualization conditions, HB-EGF evidently restrained the phosphorylation of LATS to some extent (Fig. 3b). Because of the lack of a DNA-binding domain, unphosphorylated TAZ executes its transcriptional activity by interacting with TEAD transcription factors after translocation into the nucleus^22^. After introduction of 8xGTIIC-luciferase plasmid which reflected TAZ-TEAD transcriptional activity, HB-EGF remarkably intensified the luciferase activity (Fig. 3c). We next wonder whether TAZ is involved in HB-EGF function in decidualization. After import of siRNA against TAZ, rHB-EGF failed to induce stromal cell proliferation and elevate the proportion of S-phase cells accompanied by the reduction of Ccnd3 and Cdk4 expression (Fig. 3d–h). In addition, silencing TAZ partially neutralized the mediation of HB-EGF on stromal cell differentiation, as indicated by the decline in Prl8a2 and Prl3c1 expression and ALP activity (Fig. 3i–k).

### TAZ protected uterine stromal cell differentiation from oxidative disruption

**Fig. 4:**
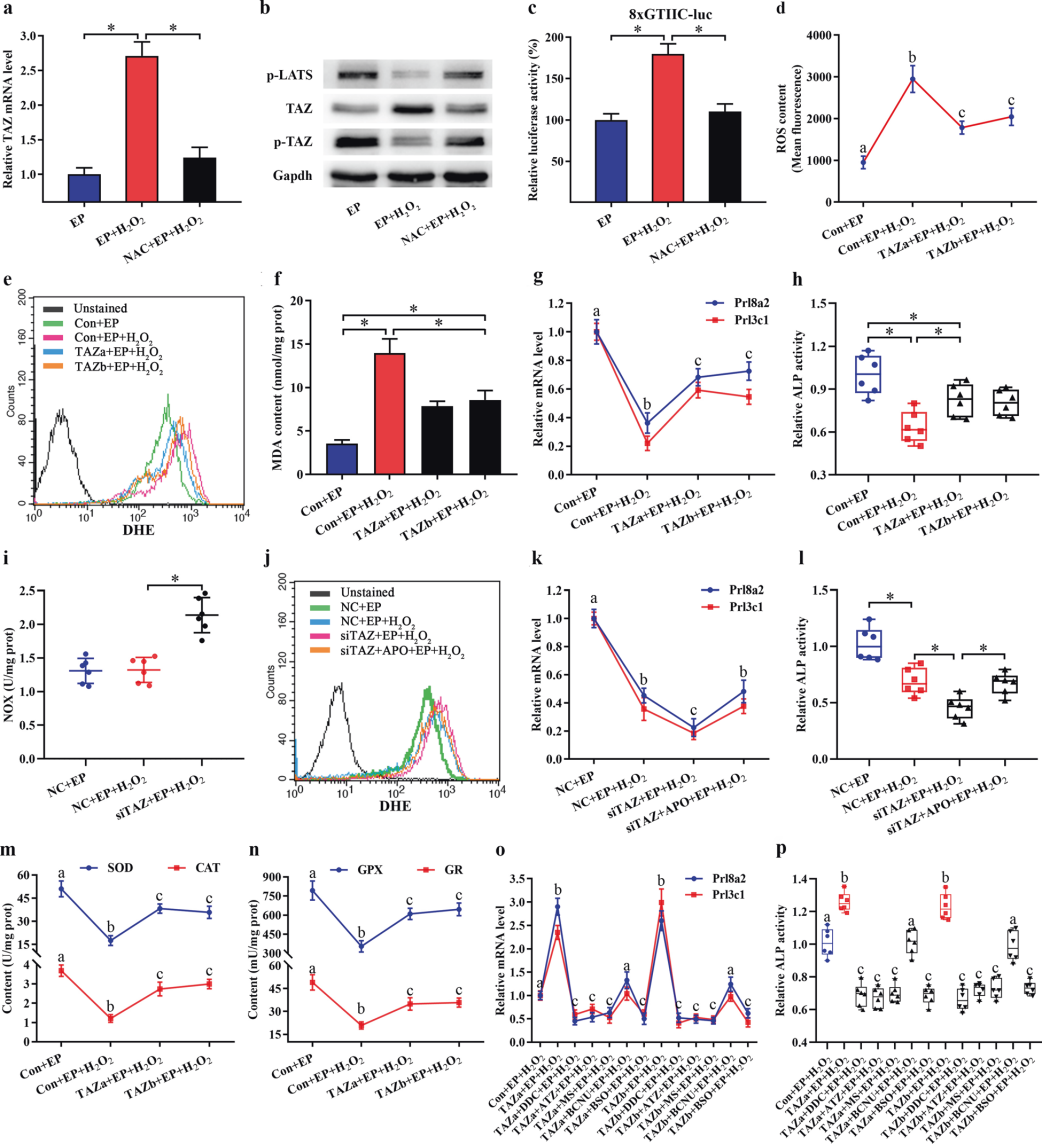
TAZ protected stromal differentiation from oxidative damage by restoring cellular antioxidant capacity. **a** TAZ mRNA expression in stromal cells after exposure to H_2_O_2_ with/without NAC. **b** TAZ and phospho-LATS protein expression after treatment with H_2_O_2_ with/without NAC. **c** TAZ-TEAD transcriptional activity after treatment with H_2_O_2_ with/without NAC. **d–f** Overexpression of TAZ attenuated the increase of intracellular ROS, O_2_^−^ and MDA levels under OS. **g**, **h** Overexpression of TAZ protected stromal cell differentiation from oxidative damage. **i** Knockdown of TAZ increased NOX activity after H_2_O_2_ exposure. **j** APO attenuated the increase of intracellular O_2_^−^ level by TAZ siRNA. **k**, **l** APO treatment reversed the repression of TAZ siRNA on stromal cell differentiation under OS. **m**, **n** Overexpression of TAZ enhanced the activities of SOD, CAT, GPX, and GR under OS. **o**, **p** Repression of SOD, CAT, GPX, and GR as well as GSH synthesis by the corresponding inhibitor blocked the protection conferred by TAZ overexpression on stromal cell differentiation under OS. Bars with different letters at the top differ significantly.

Among the many factors affecting pregnancy, OS caused by excessive ROS generation has received much attention^13,14^. Therefore, we generated an H_2_O_2_-induced OS model to investigate whether TAZ was involved in regulating the redox state of stromal cells during decidualization. After cell exposure to H_2_O_2_, TAZ mRNA and total protein levels, as well as TAZ-TEAD transcriptional activity, were markedly increased during in vitro decidualization, whereas phosphorylation of TAZ and LATS revealed a notable obvious decline (Fig. 4a–c). Supplementation of cells with ROS scavenger NAC reversed this effectiveness (Fig. 4a–c). Furthermore, overexpression of TAZ diminished intracellular ROS and O_2_^−^ levels, reduced the content of MDA, an OS indicator, and restored the expression of Prl8a2 and Prl3c1, as well as ALP activity, while knockdown of TAZ aggravated oxidative impairment of stromal cell differentiation and increased ROS, O_2_^−^ and MDA levels (Fig. 4d–h, Supplementary Fig. 3a–e).

### TAZ prevented oxidative damage to stromal differentiation by restoring cellular antioxidant capacity

Since NOX was the principal source of intracellular ROS^23^, we explored the regulation of NOX activity by TAZ. Under OS conditions, continuous expression of TAZ did not affect the NOX activity (data not shown), but blockade of TAZ enhanced the NOX activity (Fig. 4i). After transfection with siRNA against TAZ and addition of NOX inhibitor APO, intracellular O_2_^−^ level was clearly reduced accompanying by the restoration of Prl8a2 and Prl3c1 expression as well as ALP activity (Fig. 4j–l).

OS is a result of insufficient antioxidant capacity to eliminate excessive ROS^13^. After exposure to H_2_O_2_, the activities of antioxidant enzymes SOD, CAT and GPX were substantially diminished. However, sustained activation of TAZ improved above antioxidant deficiency, while downregulation of TAZ exacerbated the reduction in antioxidant enzyme activities (Fig. 4m, n, Supplementary Fig. 3f, g). The addition of SOD inhibitor DDC, CAT inhibitor ATZ or GPX inhibitor MS might block the defensive function of overexpressed TAZ on the oxidative damage to stromal cell differentiation (Fig. 4o, p). In addition, TAZ strengthened GR activity followed by the elevation of GSH content and GSH/GSSG ratio, but supplementation with GR inhibitor BCNU and GSH synthesis inhibitor BSO, that reduced GSH content and GSH/GSSG ratio, hindered the reestablishment of TAZ effects on disrupted stromal cell differentiation (Fig. 4n–p, Supplementary Fig. 3g–i, Supplementary Fig. 4a–d).

### TAZ protected the mitochondrial function of stromal cells under OS

**Fig. 5:**
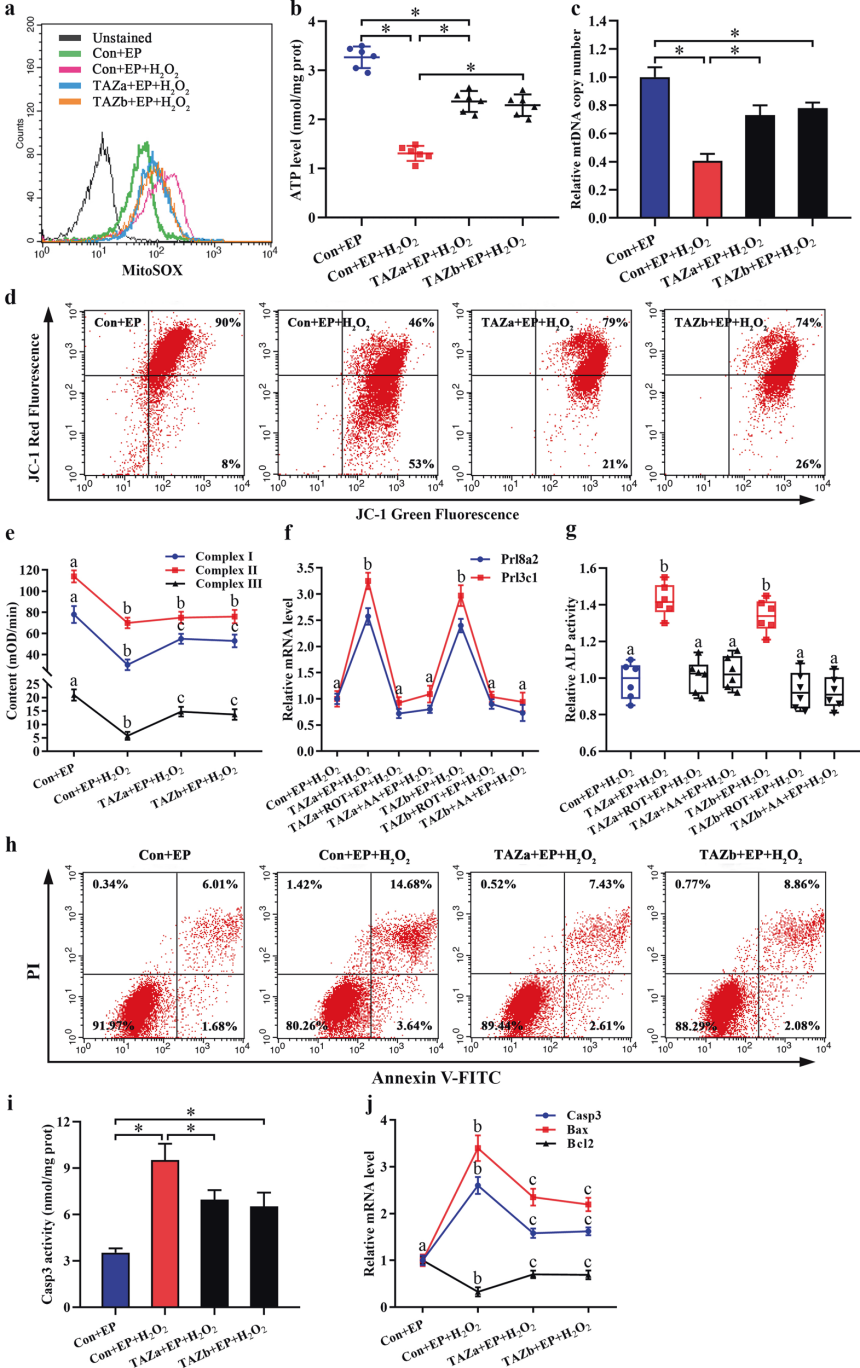
TAZ prevented mitochondrial dysfunction and stromal cell apoptosis under OS. **a** Overexpression of TAZ decreased mitochondrial O_2_^−^ level under OS. **b–d** Overexpression of TAZ hindered H_2_O_2_-induced damage to ATP level, mtDNA copy number, and mitochondrial membrane potential. **e** Effects of TAZ overexpression on the activities of mitochondrial respiratory chain complexes I, II, and III under OS. **f**, **g** ROT and AA abrogated the protection conferred by TAZ overexpression on stromal cell differentiation under OS. **h** Overexpression of TAZ blocked stromal cell apoptosis under OS. **i**, **j** Overexpression of TAZ weakened Casp3 activity, reduced the levels of Casp3 and Bax mRNA and induced the recovery of Bcl2 expression under OS.

Mitochondria are crucial for the maintenance of cellular function by governing energy production^24^. Administration of H_2_O_2_ resulted in the impairment of mitochondrial function, as evidenced by the reduction in ATP level and mitochondrial membrane potential together with aberrant mtDNA copy number and elevated mitochondrial O_2_^−^ level (Fig. 5a–d). Overexpression of TAZ prevented mitochondrial dysfunction, while knockdown of TAZ exacerbated this disorder (Fig. 5a–d, Supplementary Fig. 5a–d). Further study revealed that TAZ might modulate the activities of mitochondrial respiratory chain complexes I and III whose blockade by ROT and AA abrogated the rescue of Prl8a2 and Prl3c1 expression and ALP activity induced by TAZ overexpression, but no obvious alteration to mitochondrial respiratory chain complex II activity after sustained expression or knockdown of TAZ (Fig. 5e–g, Supplementary Fig. 5e).

### TAZ prevented H_2_O_2_ induction of stromal cell apoptosis

Previous studies have demonstrated that mitochondrial dysfunction is closely related to cell apoptosis^25–27^. In the context of H_2_O_2_, continuous expression of TAZ prevented stromal cell apoptosis, whereas depletion of TAZ heightened cell apoptosis rate (Fig. 5h, Supplementary Fig. 5f). Furthermore, overexpression of TAZ weakened Casp3 activity and decreased the mRNA levels of Casp3 and Bax, and led to the recovery of Bcl2 expression, while silencing of TAZ exhibited the opposite effects (Fig. 5i, j, Supplementary Fig. 5g, h).

### TAZ activated the Nrf2/ARE/Foxo1 pathway under OS

**Fig. 6:**
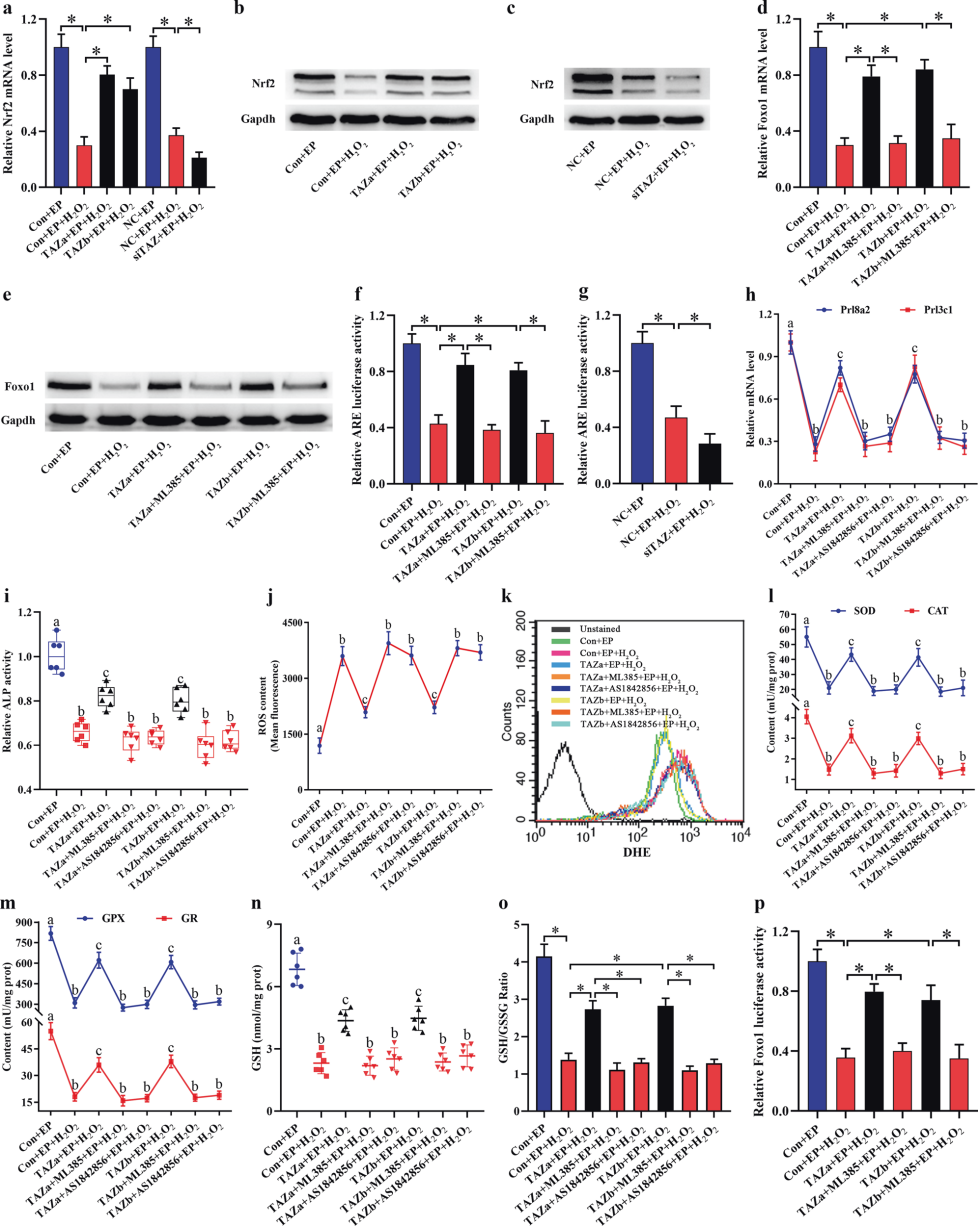
TAZ restored cellular antioxidant capacity via the Nrf2-ARE-Foxo1 pathway under OS. **a–c** Effects of TAZ overexpression or knockdown on the mRNA and protein levels of Nrf2. **d, e** Effects of TAZ overexpression on the mRNA and protein levels of Foxo1 with/without Nrf2 inhibitor ML385. **f**, **g** Effects of TAZ overexpression or knockdown on Nrf2 transcriptional activity. **h**, **i** Blockade of Nrf2 and Foxo1 with the corresponding inhibitor abrogated the protection conferred by TAZ overexpression on stromal cell differentiation under OS. **j**, **k** Repression of Nrf2 and Foxo1 impeded the reduction in intracellular ROS and O_2_^−^ levels caused by TAZ overexpression. **l–o** Blockade of Nrf2 and Foxo1 abolished the ability of TAZ to rescue the activities of SOD, CAT, GPX and GR as well as GSH content and GSH/GSSG ratio. **p** Effects of TAZ overexpression on Foxo1 promoter activity in the absence or presence of ML385.

It is well known that Nrf2 and Foxo1 are important modulators of cellular antioxidant defense^28,29^. Under OS, Nrf2 and Foxo1 expression levels, as well as Nrf2 nuclear translocation, were obviously diminished, but sustained activation of TAZ reversed these effects (Fig. 6a–e). Moreover, TAZ enhanced the transcriptional activity of Nrf2 (Fig. 6f, g). Blockade of Nrf2 and Foxo1 with the corresponding inhibitor abrogated TAZ protection of stromal cell differentiation to oxidative damage, antagonized TAZ attenuation of intracellular ROS and impeded the rescue of TAZ on the enzymatic activities of SOD, CAT, GPX and GR as well as GSH content and GSH/GSSG ratio (Fig. 6h–o). Further evidence revealed that addition of Nrf2 inhibitor ML385 abrogated the ability of TAZ to rescue the mRNA and protein levels of Foxo1 under OS (Fig. 6d, e). Analysis of Foxo1 promoter region exhibited the presence of antioxidant response element (ARE) harboring an Nrf2-binding site. Overexpression of TAZ restored the luciferase activity of Foxo1 reporter plasmid under OS, but this restoration was blocked by Nrf2 inhibitor ML385 (Fig. 6p).

## Discussion

TAZ is required for spermatogenesis and fertilization, but its physiological role in uterine decidualization has not been studied. In this study, we found that TAZ was clearly observed in decidual cells. In addition, abundant nuclear TAZ protein was identified in human endometrial stromal cells that underwent expansive proliferation and then differentiated into decidual cells, a process known as decidualization^2,12^. Stromal cell proliferation is the first step of decidualization^30^. Overexpression of TAZ drived stromal cell proliferation, while silencing of TAZ had the opposite effect. Consistently, TAZ might exert proliferative activity in mammary and periodontal ligament stem cells, and stimulate liver regeneration by increasing hepatocyte proliferation^31–33^. It is known that cellular proliferation is accompanied by the alteration of cell cycle^34^. TAZ promoted the G1/S phase transition which was adjusted by Ccnd3, a G1 phase modulator of stromal cell proliferation^2,35^. Deficiency of Ccnd3 resulted in defective decidualization concomitant with the impairment of stromal cell differentiation^2,20,35^. Constitutive activation of TAZ increased the expression or activity of Prl8a2, Prl3c1 and ALP, reliable markers for stromal differentiation, whereas knockdown of TAZ led to the opposite effects. Together these observations suggest that TAZ plays an important role in uterine decidualization through Ccnd3.

HB-EGF is a fundamental modulator of decidualization^20,21^. Ablation of HB-EGF led to compromised pregnancy outcomes with defective decidual development^20,21,36^. Adjunction of exogenous rHB-EGF facilitated the expression of TAZ total protein and diminished the phosphorylation of TAZ as well as LATS which was one of core components for Hippo pathway and its activation resulted in cytoplasmic sequestration of phosphorylated TAZ, while deletion of LATS heightened nuclear enrichment of TAZ^22,37^. Concurrently, after import into the nucleus, unphosphorylated TAZ executes its transcriptional activity by interacting with TEAD transcription factors^22^. Under in vitro decidualization conditions, HB-EGF remarkably enhanced the transcriptional activity of TAZ-TEAD. Together these data indicate that HB-EGF activates TAZ-TEAD by repressing LATS phosphorylation. Further analysis revealed that inhibition of TAZ abrogated HB-EGF induction of stromal cell proliferation and differentiation, indicating that TAZ serves as a downstream target of HB-EGF in decidualization. Previously reported data evidenced that HB-EGF might boost the expression of Ccnd3 which was necessary for HB-EGF-driven decidualization^20^. Knockdown of TAZ abolished the promotion of HB-EGF on Ccnd3, suggesting that TAZ functions as an important intermediary between HB-EGF and Ccnd3.

Intracellular ROS that was not scavenged by the intrinsic antioxidant system induced OS, which impaired decidualization^13–15^. As one of the most important cellular antioxidants^14^, GSH was shown to have diminished content under OS, while activation of TAZ improved the level of GSH whose synthesis blockade by BSO impeded the defense of TAZ against oxidative damage to stromal cell differentiation. Previous studies have shown that GPX might oxidize GSH to GSSG in metabolizing H_2_O_2_ into water, while GR diminished GSSG back to GSH^14^. In the present study, TAZ enhanced the enzymatic activities of GPX and GR, whose inhibition disrupted the protective effect of TAZ on stromal cell differentiation under OS. Together these observations suggest that TAZ may defend against oxidative damage to uterine decidualization via GSH. Furthermore, TAZ might modulate the activities of SOD and CAT which were two important antioxidant enzymes in uterine decidualization and catalyzed the dismutation of O_2_^−^ to H_2_O_2_ or the conversion of H_2_O_2_ to water^14,38^. Collectively, these results reveal that TAZ prevents oxidative damage to stromal cell differentiation by restoring antioxidant capacity.

After translocation into the nucleus, TAZ may modulate gene transcription^39^. Under OS, TAZ promoted the expression of Nrf2 which was a redox-sensitive transcription factor and its loss caused an increase in intracellular ROS due to defective antioxidant capability^40^. Repression of Nrf2 might annihilate the defense of TAZ on oxidative damage to stromal cell differentiation, indicating that Nrf2 is a downstream target of TAZ in antioxidant function. Further analysis revealed that TAZ induced nuclear translocation of Nrf2, where it might bind to the ARE of target gene promoter region^28^. Treatment with Nrf2 inhibitor ML385 abrogated the increased luciferase intensity of Foxo1 reporter plasmid induced by TAZ overexpression and impeded the TAZ stimulation of Foxo1, which was an important modulator of cellular antioxidant defense and its ablation in uteri caused infertility owing to the alteration of epithelial integrity along with aberrant decidual response^29,41^. Moreover, blockade of Foxo1 abolished the ability of TAZ to defend stromal cell differentiation from oxidative disruption which was followed by the enhancement of intracellular ROS levels. Together these observations suggest that TAZ activates the Nrf2/ARE/Foxo1/ROS pathway to protect stromal cell differentiation against oxidative damage.

NOX is the principal source of intracellular ROS^23^. Attenuation of TAZ led to an obvious elevation in NOX activity along with abundant accumulation of intracellular ROS. Impediment of NOX by APO attenuated the damage to stromal cell differentiation elicited by TAZ knockdown concomitant with the reduced O_2_^−^ level, indicating the importance of TAZ in mediating ROS sources via NOX. In addition, as crucial organelles for energy production, mitochondria generate ROS, but excessive ROS can attack mitochondria, resulting in mitochondrial dysfunction as evidenced by the reduction in ATP synthesis, mitochondrial membrane potential and respiratory enzyme activities along with the elevation of mitochondrial ROS levels^25,26,42^. Under OS conditions, TAZ prevented mitochondrial dysfunction in stromal cells. Furthermore, mtDNA is vulnerable to excessive ROS owing to the lack of protective histones^25,26^. After exposure to H_2_O_2_, TAZ ameliorated oxidative damage to mtDNA copy number, reinforcing the role of TAZ in protecting mitochondrial function. Further analysis showed that mitochondrial dysfunction caused cell apoptosis which was mediated by the apoptosis executor Casp3, pro-apoptotic effector Bax and anti-apoptotic Bcl2^25–27^. During in vitro decidualization, TAZ prevented the apoptosis of stromal cells induced by OS through raising Bcl2 expression and suppressing the upregulation of Casp3 and Bax.

**Fig. 7:**
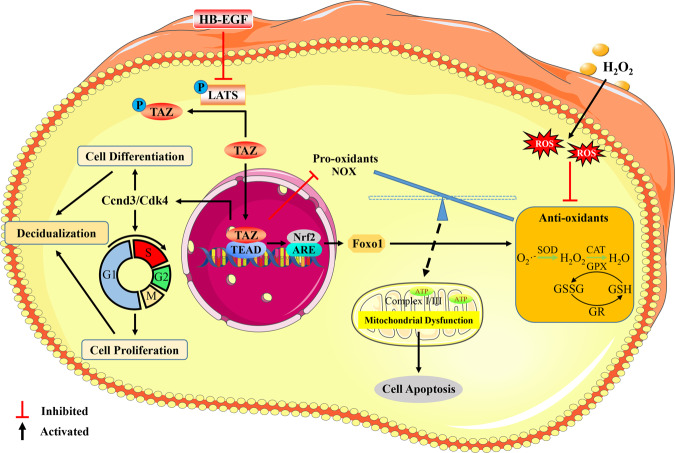
Schematic depiction of TAZ function and regulation in decidualization. TAZ might exert an important role in uterine decidualization via Ccnd3 and Cdk4 responsiveness to HB-EGF. Furthermore, TAZ ameliorated oxidative damage to stromal differentiation by enhancing cellular antioxidant capacity dependent on Nrf2/ARE/Foxo1 pathway, restoring mitochondrial function and inhibiting cell apoptosis.

In conclusion, TAZ might play an important role in uterine decidualization via Ccnd3 and Cdk4 in response to HB-EGF. Furthermore, TAZ ameliorated the oxidative damage to stromal cell differentiation by enhancing antioxidant capacity dependent on the Nrf2/ARE//Foxo1 pathway, recovering mitochondrial function and repressing cell apoptosis (Fig. 7).

## Supplementary information

Supplementary Material

## Data Availability

The data that support the findings of this study are available on request to the corresponding author.
