# Supplementary material for: TAZ as a novel regulator of oxidative damage in decidualization via Nrf2/ARE/Foxo1 pathway
Source: Exp Mol Med. 2021 Sep 8;53(9):1307–18. doi: 10.1038/s12276-021-00655-2 (PMC8492733; doi:10.1038/s12276-021-00655-2)
Supplement: Supplementary file 1 — Supplementary Material [file 12276_2021_655_MOESM1_ESM.doc]

**Supplementary Figure 1**

**
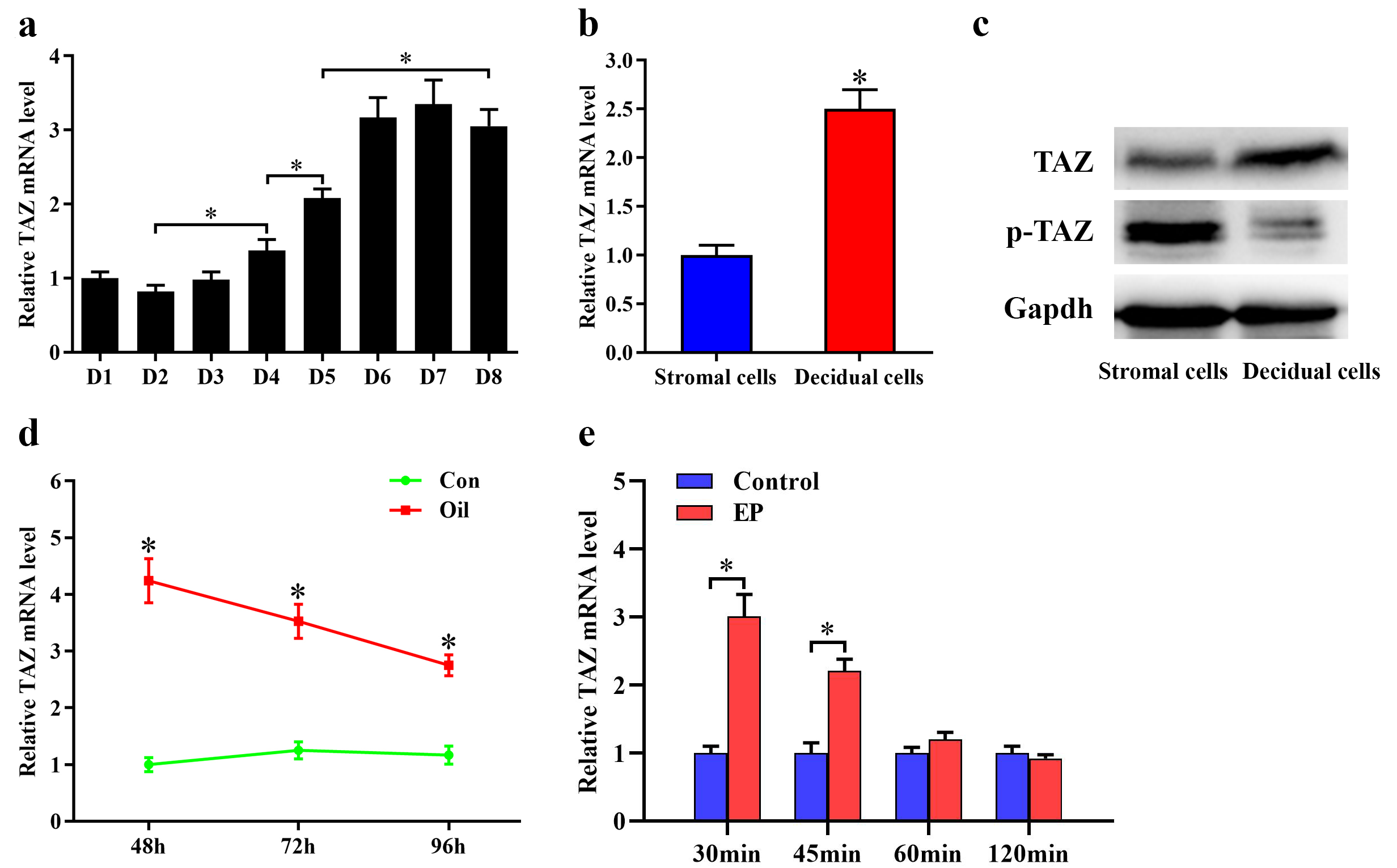
**

**Supplementary Figure 1. Real-time PCR and western blotting** **analyses of TAZ expression in uteri and decidual cells. a** TAZ mRNA in uteri during early pregnancy. **b, c** TAZ mRNA and protein levels in decidual and stromal cells. **d** TAZ mRNA under artificial decidualization. Con, uninjected uterine horn served as a control; Oil, oil-induced decidualization. **e** TAZ mRNA during in vitro decidualization. EP, estrogen plus progesterone.

**Supplementary Figure 2**


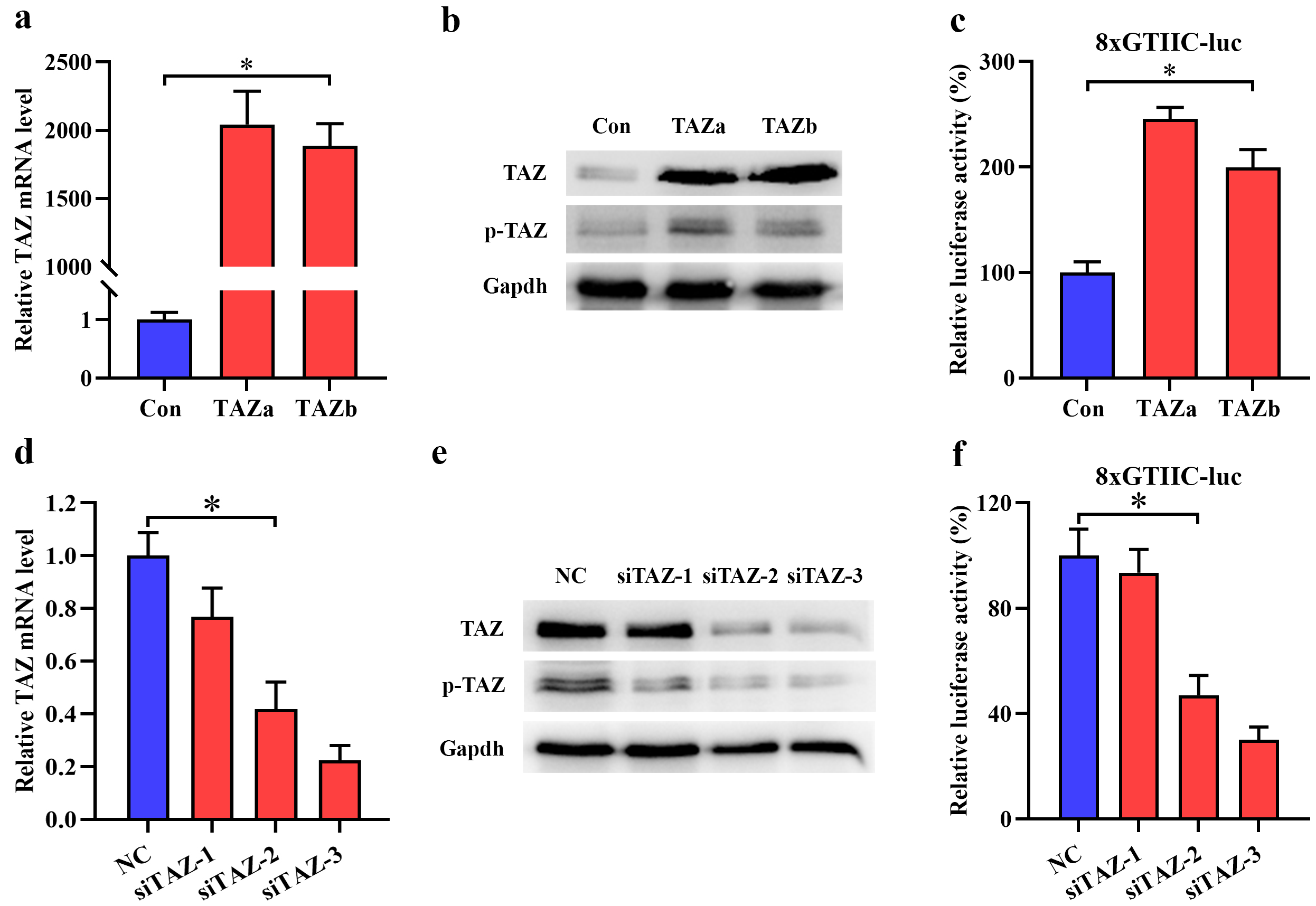


**Supplementary Figure 2. TAZ mRNA and protein levels as well as TAZ-TEAD transcriptional activity in stromal cells after transfection with TAZ overexpression plasmid or siRNA. a-c** TAZ mRNA and protein levels as well as TAZ-TEAD transcriptional activity in stromal cells after introduction of TAZ overexpression plasmid for 48 h. **d-f** TAZ mRNA and protein levels as well as TAZ-TEAD transcriptional activity after introduction of TAZ siRNA for 48 h.

**Supplementary Figure 3**

**
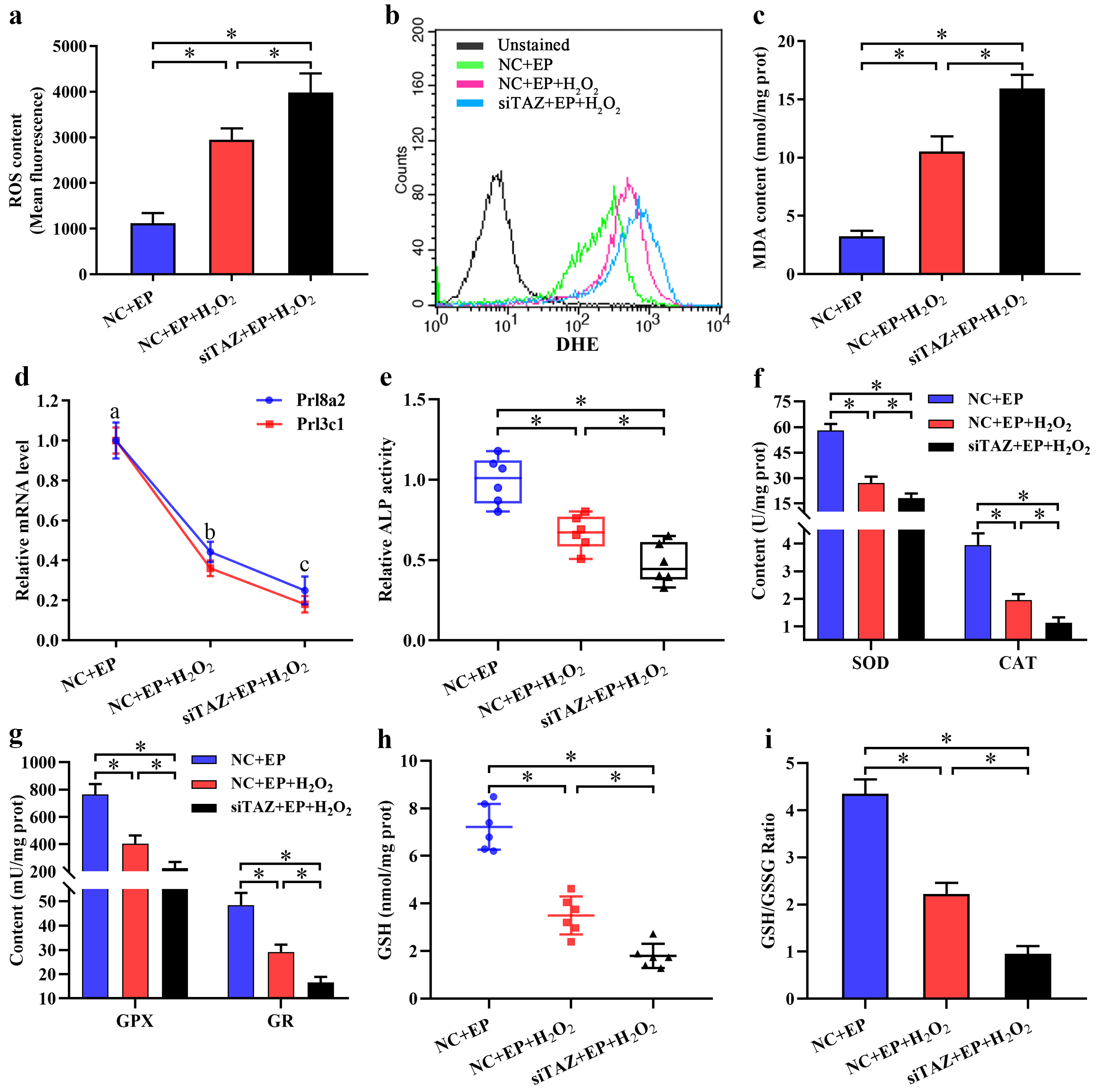
**

**Supplementary Figure 3. Silencing of TAZ aggravated oxidative impairment to stromal differentiation with the elevation of intracellular ROS and defective antioxidant capacity under OS. a-c** Silencing of TAZ increased intracellular ROS, O_2_^−^ and MDA levels after exposure to H_2_O_2_. **d, e** Knockdown of TAZ aggravated oxidative impairment to stromal differentiation under OS. **f, g** Knockdown of TAZ exacerbated the reduction of SOD, CAT, GPX and GR activities under OS. **h, i** Knockdown of TAZ diminished GSH content and GSH/GSSG ratio under OS.

**Supplementary Figure 4**


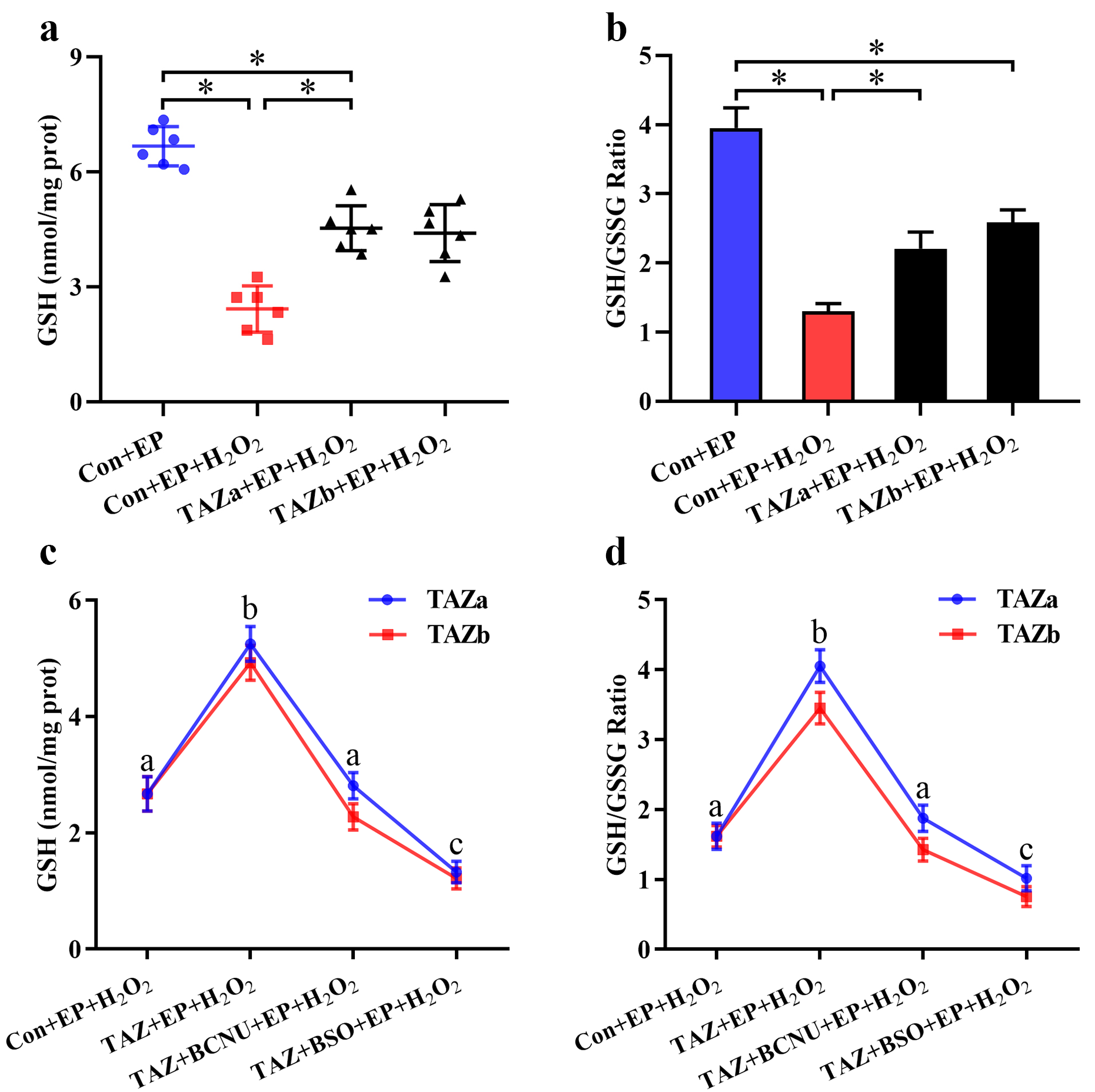


**Supplementary Figure 4. Blockage of GR and GSH synthesis impeded the rescue of TAZ overexpression on GSH content under OS. a, b** Overexpression of TAZ strengthened GSH content and GSH/GSSG ratio. **c, d** Suppression of GR and GSH synthesis by BCNU or BSO neutralized the enhancement of TAZ on GSH content and GSH/GSSG ratio.

**Supplementary Figure 5**

**
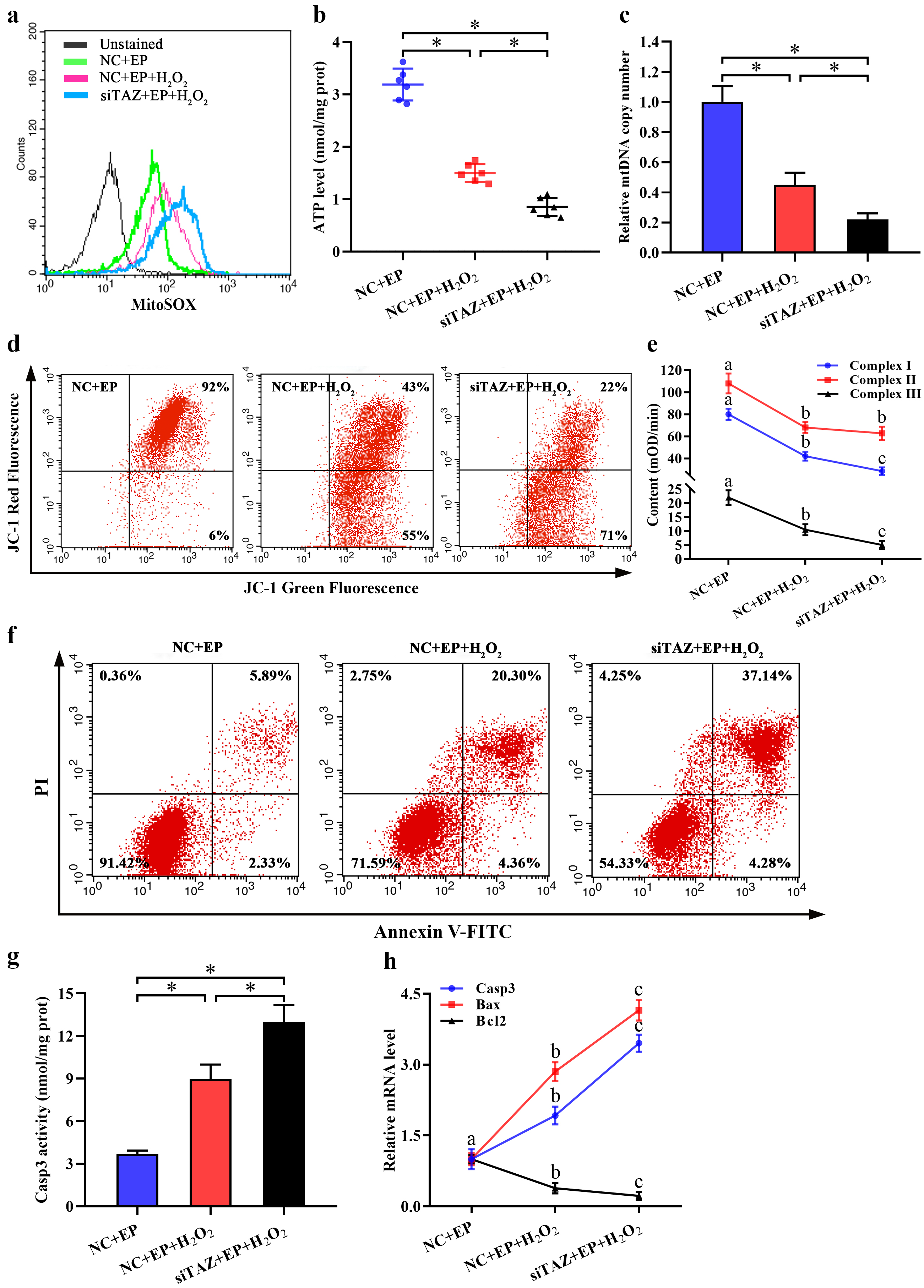
**

**Supplementary Figure 5. Effects of TAZ knockdown on mitochondrial function and stromal cell apoptosis under OS. a** Knockdown of TAZ increased mitochondrial O_2_^−^ level. **b-d** Knockdown of TAZ deteriorated the impairment of H_2_O_2_ to ATP, mtDNA copy number and mitochondrial membrane potential. **e** Effects of TAZ siRNA on the activities of mitochondrial respiratory chain complexes I, II and III. **f** Depletion of TAZ heightened stromal cell apoptosis. **g, h** Silencing of TAZ increased Casp3 activity followed by the elevated mRNA levels for Casp3 and Bax, and decreased Bcl2 expression.

Supplementary Table 1. Primers used in this study

| Gene | Sequence of Forward Primer | Sequence of Reverse Primer | Application |
| --- | --- | --- | --- |
| TAZ | CAGTAGCTCAGATCCTTTCC | TGATGTAGAGTCTGCTCTGA | In situ hybridization |
| TAZ | CGACTCAGAACCAACCCACA | CATGAGCTCCTCTTGACGCA | Real-time PCR |
| Nrf2 | CCCAGCACATCCAGACAGAC | TATCCAGGGCAAGCGACTCA | Real-time PCR |
| Foxo1 | GAAGAGCGTGCCCTACTTCAA | GATTGAGCATCCACCAAGAACT | Real-time PCR |
| TAZa | GATATCTGGCTCCGGCCAGGAGGTCAT | CTCGAGGTGATTACAGCCAGGTTAGA | Overexpression |
| TAZb | GATATCCCGAGTCCCCAGAAAGATG |
